# Consumer Data is Key to Artificial Intelligence Value: Welcome to the Health Care Future

**DOI:** 10.2196/68261

**Published:** 2025-08-01

**Authors:** James P Cummings

**Affiliations:** 1 Participatory Health, 20 Grasmere AveFairfield, CT, 06824, United States, 1 (212) 280-1600

**Keywords:** consumer data, artificial intelligence, longitudinal health records, data interoperability, large language models, health IT standards, policy regulation, rare disease registries, translational science, precision medicine, patient participation, genomic and wearable data, nodal graph architecture, FHIR, 21st Century Cures Act, resilient systems, Fast Healthcare Interoperability Resource

## Abstract

Humanity stands at the threshold of a new era in biological understanding, disease treatment, and overall wellness. The convergence of evolving patient and caregiver (consumer) behaviors, increased data collection, advancements in health technology and standards, federal policies, and the rise of artificial intelligence (AI) is driving one of the most significant transformations in human history. To achieve transformative health care insights, AI must have access to comprehensive longitudinal health records (LHRs) that span clinical, genomic, nonclinical, wearable, and patient-generated data. Despite the extensive use of electronic medical records and widespread interoperability efforts, current health care organizations, electronic medical record vendors, and public agencies are not incentivized to develop and maintain complete LHRs. This paper explores the new paradigm of consumers as the common provenance and singular custodian of LHRs. With fully aligned intentions and ample time to dedicate to optimizing their health outcomes, patients and caregivers must assume the sole responsibility to manage or delegate aggregation of complete, accurate, and real-time LHRs. Significant gaps persist in empowering consumers to act as primary custodians of their health data and to aggregate their complete LHRs, a foundational requirement for the effective application of AI. Rare disease communities, leaders in participatory care, offer a compelling model for demonstrating how consumer-driven data aggregation can be achieved and underscore the need for improved policy frameworks and technological tools. The convergence of AI and LHRs promises to transform medicine by enhancing clinical decision-making, accelerating accurate diagnoses, and dramatically advancing our ability to understand and treat disease at an unprecedented pace.

## Introduction

Artificial intelligence (AI), particularly large language models (LLMs), holds extraordinary promise in unlocking and interpreting vast volumes of data for public and professional use. Of the projected 180 Zettabytes [1] of global data to be collected by 2025, less than 1% is ever tagged or analyzed [2]. Without the aid of AI, the sheer volume of data exceeds human capacity to meaningfully use this valuable collection.

Health care represents one of the most data-intensive sectors, contributing one-third of the world’s data through electronic medical records (EMRs), imaging technologies, genomics, and personal devices [3]. LLMs offer unprecedented capabilities to process this magnitude of data. Untiring and limitless, LLMs’ computational power liberates the human burden of manual analysis to unlock intellectual and creative potential. The ability of AI to expedite biomedical discovery advancement is profound and exciting.

However, it is important to acknowledge AI will only ever access a small fraction of the global data landscape. Currently, less than 3% [2] of the world’s data is openly accessible; the vast majority remains proprietary, restricted, or siloed within the organizations that generated it.

To realize the full potential of AI in health, models must be trained on rich, proprietary, real-time datasets. This paper outlines the pathway and necessary processes for LLMs to ingest and learn from comprehensive, longitudinal health data—empowering consumers, transforming personalized care, and increasing biological and disease understanding by a significant measure.

## AI Appetite

Foundational LLMs, such as GPT-40 [4], Claude [5], or Llama [6] are trained on open data commons, including papers, articles, books, websites, and others from sources, such as Common Crawl [7], Wikipedia [8], PubMed [9], Data.gov [10], World Digital Library [11], GitHub [12], and so on. LLMs use these large data stores to explain, assist, and suggest solutions to user prompts. Open data repositories are continuing to grow and become more frequently used. However, privacy, security risks, and commercial interests remain significant factors in LLMs’ accessibility to data. The value of LLMs for health is inextricably tied to the quantity and quality of the data on which it is trained and the proprietary data on which it is fed.

## Data Gold

Organizations tag and analyze vast volumes of proprietary data to enhance efficiency, manage risk, improve customer experience, and maintain a competitive advantage. Manufacturing, finance, telecommunication, eCommerce, and health care are the most significant contributors to the world’s data. AI models enable organizations to maximize the use of their raw data. By feeding LLMs comprehensive custom data, entities can harness their proprietary datasets to uncover novel insights to make optimal strategic decisions.

## Health Data

Epic Systems [13] is the world’s largest EMR vendor, followed by Oracle Health (formerly Cerner) [14], MEDITECH [15], and Veradigm (formerly Allscripts) [16]. EMRs encompass patient demographics, medical history, clinical notes, medications, lab results, radiology reports, procedures, and other therapeutic data.

Epic holds at least 1 record for nearly 94% [17] of Americans; over 325 million US citizens have a health record in Epic [18]. Their market share grew from 31% in 2021 [19] to 37.7% in 2024, adding 153 new hospitals and now covering over half of acute care multispecialty beds nationwide [20]. In addition to enterprise and billing capability, Epic’s strengths include a comprehensive platform with a focus on interoperability, continuity of care, and ability to integrate with a variety of information technology systems. Epic’s “Care Everywhere” platform enables patient health information exchange across multiple provider organizations and EMR systems. Providers can query other health systems connected through “Care Everywhere” and import patient data into their EMR with aims of enhancing care, reducing medical errors, and improving patient outcomes.

Epic’s popularity is also due to their patient portal, MyChart, which addresses the growing patient demand of taking an active role to manage their health and meet the requirements of the 21st Century Cures Act [21]. MyChart provides patients access to their medical records, appointment scheduling, secure messaging, bill pay, prescription management, telehealth capability, and wellness tracking.

## Pivotal Policy

As health data collection and consumer participatory behavior grows, so does the need for policies fostering greater data exchange and accessibility. The 21st Century Cures Act, signed into law by President Obama on December 13, 2016, promotes health interoperability and gives patients access and more control over health data.

Since the Cures Act signing, the Assistant Secretary for Technology Policy (ASTP)/Office of the National Coordinator for Health Information Technology (ONC) [22] introduced several provisions, which mandate for interoperability, patient access to health information, data privacy and security, information blocking prevention, and application programming interface requirements to access data.

The United States Core Data for Interoperability (USCDI) [23], first released by the ASTP in July 2020, are foundational standards that define minimum data elements for national data exchange across health systems. USCDI defines specific categories of health information (data classes) and essential data elements within those categories. These include patient demographics (name, date of birth, address, and contact information), clinical information (allergies, medications, immunizations, laboratory test results, and vital signs), and care coordination (clinical notes, goals, and health concerns).

## Necessary Technology

A crucial component of contemporary health data policy is the adoption of Fast Healthcare Interoperability Resource (FHIR), one of the most significant advancements in EMR data exchange [24]. Developed and maintained by Health Level 7 International (HL7), FHIR is an open, license-free standard that is publicly available and designed to promote seamless interoperability. FHIR builds upon its predecessor, the HL7 Consolidated Clinical Document Architecture (C-CDA) [25], a document-based standard used to capture a “point-in-time” snapshot of a consumer health record.

Unlike C-CDA, FHIR uses modern web technology, such as RESTful APIs, JSON, and XML to enable consistent data exchange. Importantly, FHIR APIs allow for “real-time” data exchange through their discrete resource design. Unlike other standards, FHIR modular structure enables users to query specific granular data elements, rather than entire documents, offering greater precision and instant access to patient records. On April 5, 2021, the ASTP enacted rules mandating FHIR-based APIs, requiring health IT developers, EMR vendors, and health care providers to adopt standard-based APIs using FHIR to access and share health data efficiently. FHIR adoption is rapidly expanding globally, with strong support from governments, international health organizations, and technology vendors. With FHIR, patients can finally retrieve their data on demand, giving them greater control over their medical information—realizing a key objective of the 21st Century Cures Act.

## Lone Custodian

Health data has historically been managed by health care organizations through their Health Information Management (HIM) departments and controlled by the EMR vendors. The quality, accuracy, and completeness of a patient’s record have largely depended upon the capabilities and priorities of these institutional custodians.

A 2010 study found that the average patient will see 28.4 different providers over their lifetime [26]. As consumer participation evolves with patients seeking alternative treatment pathways and second opinions, this number is likely to grow. As a result, patient data becomes more scattered across different health systems and EMR platforms.

Meanwhile, a vast amount of valuable nonclinical patient-contributed data (PCD), often not collected in clinical settings, remains outside EMR. This includes data from genomic sequencing, wearable trackers, social determinants of health, remote monitoring devices, patient-reported outcomes, lifestyle data, such as nutrition and fitness, behavioral health factors, family history, symptomatic, mobile app logs, and patient-generated data. Far more abundant than the clinical data captured in EMRs, PCD, much of which is in the possession of the consumer, is increasingly recognized as essential for personalized care, prevention, wellness, and chronic disease management. Studies continue to confirm the importance of health data not included in the EMR [27].

**Figure 1.**
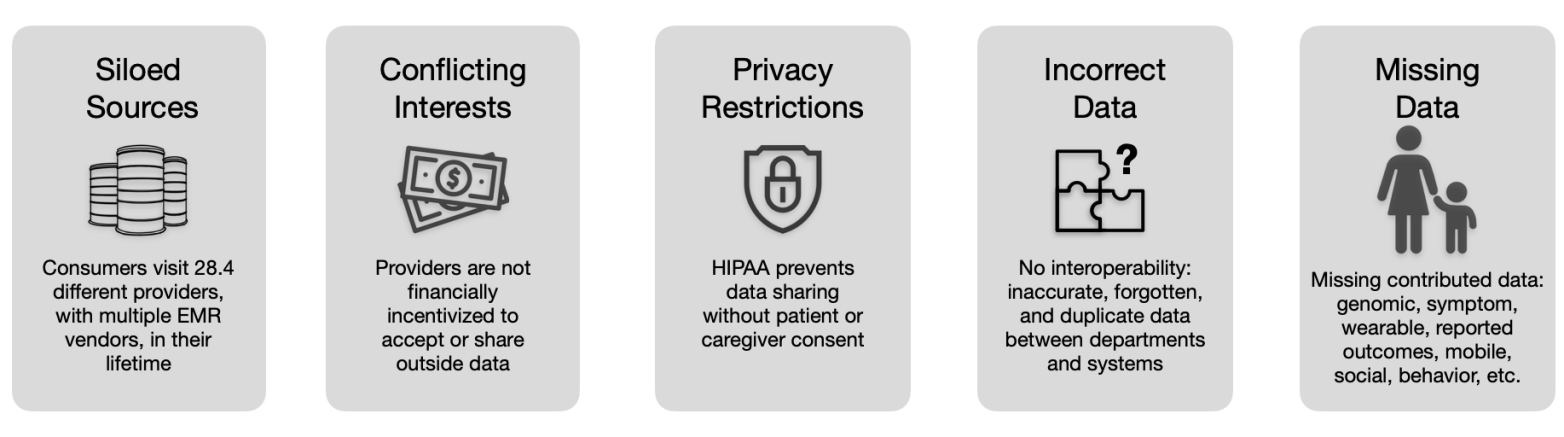
Health care system challenges to a longitudinal health record. EMR: electronic medical record; HIPAA: Health Insurance Portability and Accountability Act.

No single health care provider, health care system, or EMR vendor can maintain a complete longitudinal health record (LHR) for a consumer. With HIPAA (Health Insurance Portability and Accountability Act) privacy laws and institutional limitations (Figure 1), health care systems are neither motivated nor incentivized to aggregate, manage, and maintain a comprehensive LHR particularly if data includes genomic, nonclinical, PCD, and wearable data. Regardless of technical capability and well-equipped EMRs, health care organizations will never assume the role of custodian for complete LHRs.

Instead, the consumer is emerging as the only viable steward of their full longitudinal health data—uniquely positioned to manage, integrate, and share across clinical and nonclinical domains. LHRs, which include all USCDI-tagged medical data along with nonclinical and personal health information, will prove immensely valuable for managing and optimizing individual and population outcomes. The consumer is, and will always be, the only custodian of the complete and real-time personal LHR. As such, the aggregation or delegation of their LHR plays a crucial role in empowering providers to apply their expertise more effectively.

## Time and Intent

Despite their education, deep experience, and oath to provide the best possible care, clinicians face constraints: limited time, health care organization incentives, and competing demands. Though no fault of their own, they are overworked and undersupported, juggling large patient loads, administrative duties, fiduciary responsibilities, malpractice concerns, and the pressures of their personal lives. Throughout history, medical professionals have increased the quality and longevity of humans’ lifespan through advances in epidemiology, microbiology, vaccination, imaging, molecular biology, therapeutics, such as antibiotics, organ transplantation, and stem cell and gene-based treatments.

For centuries, patients have been entirely dependent on clinicians’ knowledge and ability to diagnose and treat disease. The paternalistic relationship excluded consumers from any meaningful role in their health between encounters. Patient expectations were shaped solely on the last clinical encounter or lab test results and remained unchanged until the next visit.

## New Paradigm

**Figure 2.**
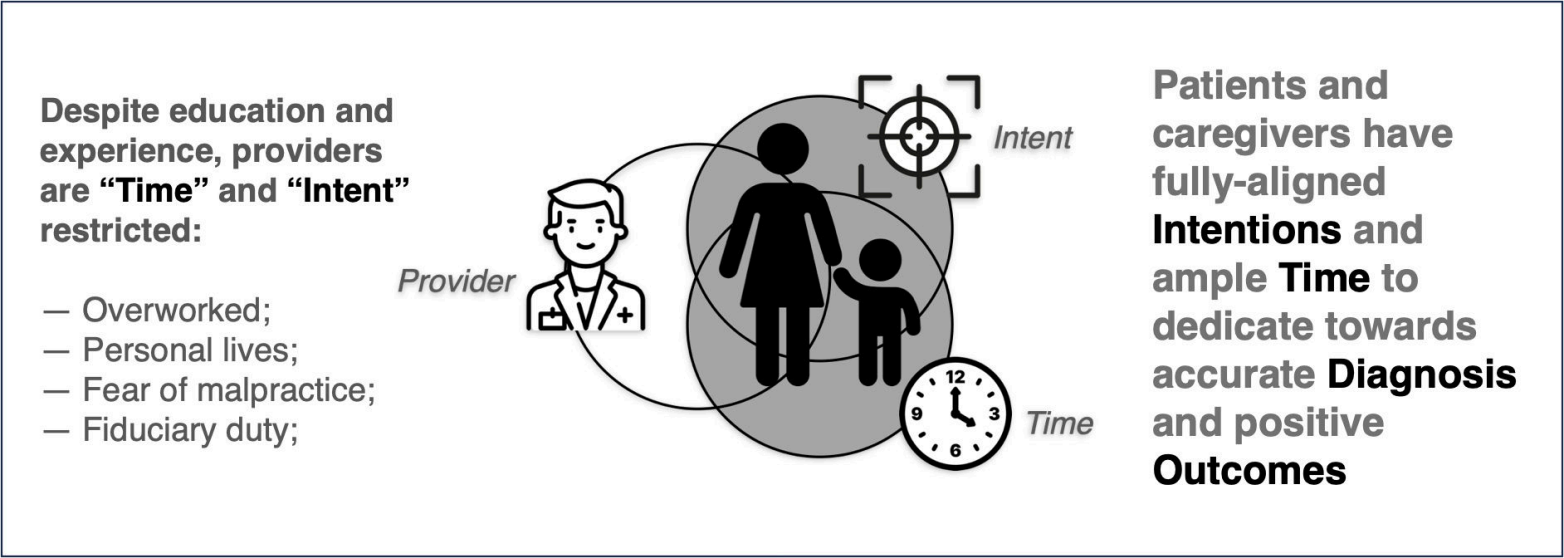
Contextual factors contributing to participatory care.

Patients and caregivers live with their disease and symptoms 24/7. They have ample time and fully aligned intentions to dedicate to achieving better outcomes. Though patients have no formal medical training or experience, their engagement is invaluable. With access to the same information as medical students and practitioners, consumers can use their time and intent to enhance their health literacy. Open forums, such as PubMed, Google, and Facebook (Meta) groups provide generous helpings of information for consumers to absorb. This knowledge positively impacts face-to-face encounters and demonstrates participatory care, helping achieve accurate diagnoses and optimal treatment outcomes. As consumers increasingly realize their value, active participatory patient behavior will become more universal and forever change the practice of medicine (Figure 2).

## Principal Responsibility

As health care evolves beyond paternalistic models, the intensity and frequency of consumer participation will naturally vary based on individual cognitive capacity, creativity, and motivation. However, regardless of the consumer’s level of engagement, or lack thereof, they or their caregiver have the sole administrative responsibility to aggregate and manage their health data. At every state of health and disease, assembling a comprehensive and complete personal LHR will always be consumers’ most basic role. While advances in technology, government policy, legacy health care systems, and 3rd party application developers improve data aggregation capabilities, the enduring burden to maintain a complete, accurate, and up-to-date LHR rests with the consumer.

**Figure 3.**
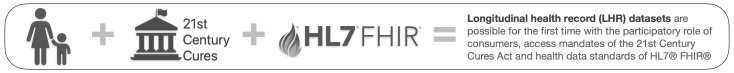
Patient and caregiver participation is crucial, along with government policy and technology standards, to assembling longitudinal health records. FHIR: Fast Healthcare Interoperability Resources; HL7: Health Level 7.

Innovations in data exchange, driven by evolving consumer behavior, ASTP policy, and FHIR standards have introduced the new paradigm of a singular, unified LHR (Figure 3).

**Figure 4.**
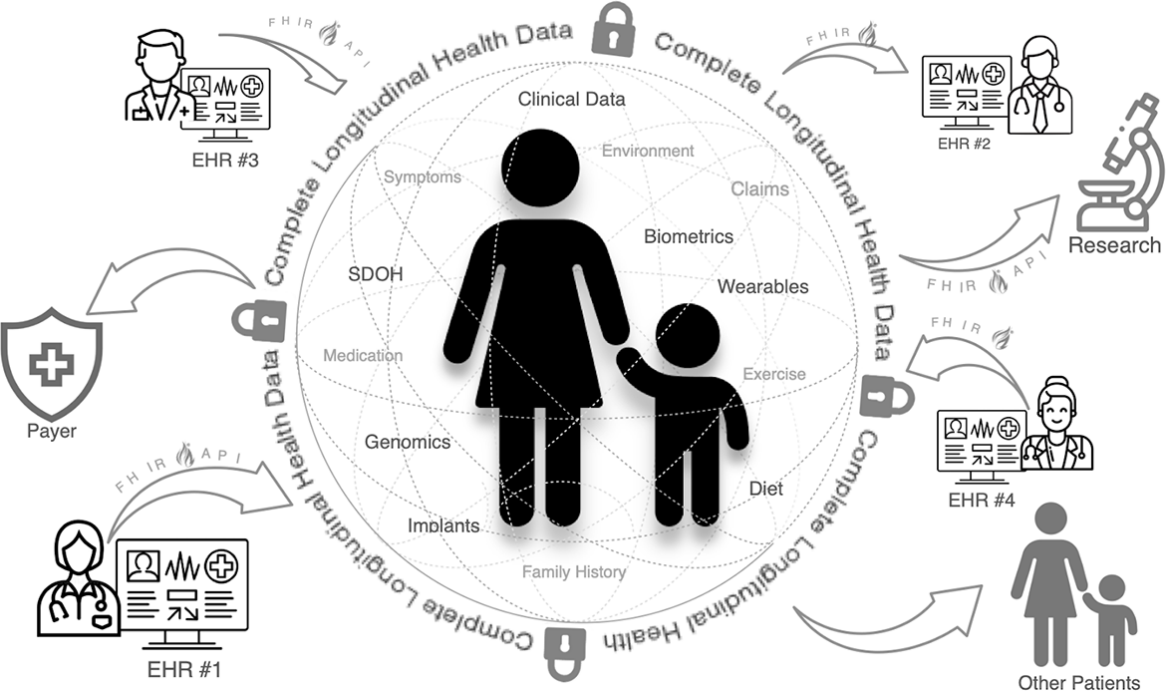
The consumer is the sole aggregating custodian of longitudinal health records consisting of multiple clinical providers and electronic medical records vendors and patient-contributed data, including genomic, mobile, symptom, behavior, and wearable data. API: application programming interfacing; EHR: electronic health record; FHIR: Fast Healthcare Interoperability Resource; SDOH: social determinants of health.

For the first time, it is technically possible for patients to consolidate all their EMR, genomic, nonclinical, and wearable data into a central repository for analysis or sharing. Access to complete LHRs allows providers to use their expertise to achieve more positive outcomes. This shift has profound implications. Most importantly, the integration of LHRs with LLMs unlocks the ability to synthesize and interpret exhaustive and up-to-date patient health histories. Given the time and cognitive limitations of humans (clinicians, patients, and caregivers), such analysis would not be feasible without LLMs—making LHRs plus AI essential partners for personalized precision care (Figure 4).

## Forever On-Call

While GPT-4 may lack clinical intuition or the real-world experience physicians develop over time, its usefulness is undeniable. Open AI GPT-4 scored 90% [28] on the United States Medical Licensing Examination (USMLE), surpassing the minimum passing threshold for medical students and demonstrating strong medical knowledge application.

AI and LLMs augment clinicians’ education and experience and have the potential to enhance clinical decision-making. LLMs facilitate knowledge extension, enabling general practitioners to deepen their understanding of specialty areas and encourage specialists to consider broader perspectives beyond their domain expertise. In doing so, LLMs promote more integrative care.

A growing number of use cases show LLMs’ capability to support diagnostic accuracy and treatment recommendations, particularly when provided with LHR datasets. One compelling example involves Courtney Morales Hoffman, the mother of a child who remained undiagnosed after seeing 17 different providers. Her son lost mobility and was failing to thrive, so she turned to GPT-4 for answers [29]. After inputting his health data, she received a possible diagnosis: Tethered Spinal Cord Syndrome. Her son’s physician confirmed the diagnosis, leading to corrective surgery. Today, her son is a healthy boy.

Human processing capacity is limited, but LLMs are always available, operating 24/7. They are tireless medical consultants for both clinicians and consumers seeking clarity, analysis, explanation, and insight. LLMs can offer diagnoses and recommend treatments when fed comprehensive LHR datasets. LHR-fed LLMs are poised to revolutionize biomedical research, accelerating discovery and improving outcomes.

## Real-World Catalysts

Undiagnosed patients and caregivers like Courtney and her son understand the importance of managing their LHR data and leveraging LLM tools in search of answers. Similarly, rare disease communities, often underfunded and underserved, have long accepted and embraced their participatory responsibility in the absence of formal support.

Patients and caregivers, particularly those navigating and managing congenital or genetic conditions, quickly take proactive roles early in their diagnostic and care journey. These rare disease consumers are more than willing to participate in the limited existing research, grant unfettered access to their EMR data, and often take the lead in fundraising efforts.

A disease is classified “rare” when it affects fewer than 1 in 2000 individuals. Extremely rare diseases are considered ultra-rare if less than 1 in 50,000 are impacted, while hyper-rare conditions occur in fewer than 1 in 100,000,000 people [30]. Some nano-rare diseases are so uncommon that their mutations are unique to a single patient or known to affect fewer than 30 people [31].

## Motivated to Share

Rare and undiagnosed patient communities offer demonstrative proof of how consumers manage and share health data in ways that significantly differ from other areas, such as finance, politics, religion, or shopping and commerce. Quite the contrary, when facing mortality, data sharing becomes a survival strategy, and consumers willingly exchange privacy for insights in hopes of benefiting from more accurate diagnoses and better outcomes.

Most rare diseases are genetic in origin [32], and those living with them are among the most motivated health consumers. The rarer the disorder, the less likely patients will find expertise among their care team. As a result, patients and families often seek out the few specialists and researchers worldwide who focus on their specific orphan disease.

The most highly engaged patients and caregivers are those managing severe, chronic, congenital genetic diseases—particularly those with ultra- and hyper-rare categories. When parents and caregivers learn about their child’s life-altering condition, they embrace an elevated responsibility, despite lacking formal medical training or experience. With aligned purpose and ample time, these communities significantly contribute to advancing critical understanding of the human genome and accelerating therapeutic discoveries.

Their efforts exemplify the promise of precision medicine. The study and tailored treatment of severe rare genetic diseases, driven by highly engaged, data-sharing consumers, represent the apotheosis of precision medicine and a roadmap for future advancements in biological understanding.

## Shining Example

With little attention and funding for research, and no “magic” doctor or treatment, patients with rare diseases recognize their fundamental responsibility and critical contribution in advancing medical understanding by aggregating and consenting to share their health data. Investigators leading rare disease registries [33] rely heavily on this active patient participation to apply observational methods to expand disease knowledge.

For example, the Cystic Fibrosis Foundation Patient Registry (CFFPR), one of the largest congenital severe rare diseases [34], has operated since 1966. This registry actively engages patients to collect and contribute comprehensive clinical and genomic data to support collaboration with clinicians and researchers. Similarly, registries like DuchenneConnect, focused on Duchenne muscular dystrophy [35], now incorporate nonclinical and wearable technology data to track mobility, physical activity, and respiratory function.

As registries evolve to include exponentially growing sources of nonclinical data, such as wearable trackers, social determinants of health, monitoring devices, patient-reported outcomes, nutrition, fitness, behavioral metrics, family history, symptoms, and mobile health apps—they can become powerful platforms to produce real-world LHR datasets that complement and extend far beyond EMRs alone.

## Powerfully Rare

The research funding and attention secured by a single severe congenital genetic rare condition are minuscule compared with support for common conditions, such as heart disease or cancer. However, despite individual rarity, rare diseases collectively affect over 10% of the US population—approximately 30 million people [36]. In 2019, the collective economic implications of rare diseases in the United States reached nearly US $1 trillion, making it one of the largest burdens on our health care system [37].

Today’s environment, shaped by the 21st Century Cures Act, technology standards like FHIR APIs, and the motivated behavior of rare disease consumers, affords an optimal ideal landscape to pilot the collection of complete LHRs across an entire disease registry cohort. Equipping researchers with full LHR datasets for all registry participants would confirm the consumer-led data aggregation model and demonstrate the maximum potential benefit of AI in medicine. The most vital and immediate need to help this new paradigm is to design and refine an LHR aggregation infrastructure for one or several congenital genetic rare disease registries. This prototype can then be replicated across the broader rare disease ecosystem—and ultimately benefit all consumers.

## AI-Enabled LHR Integration

**Figure 5.**
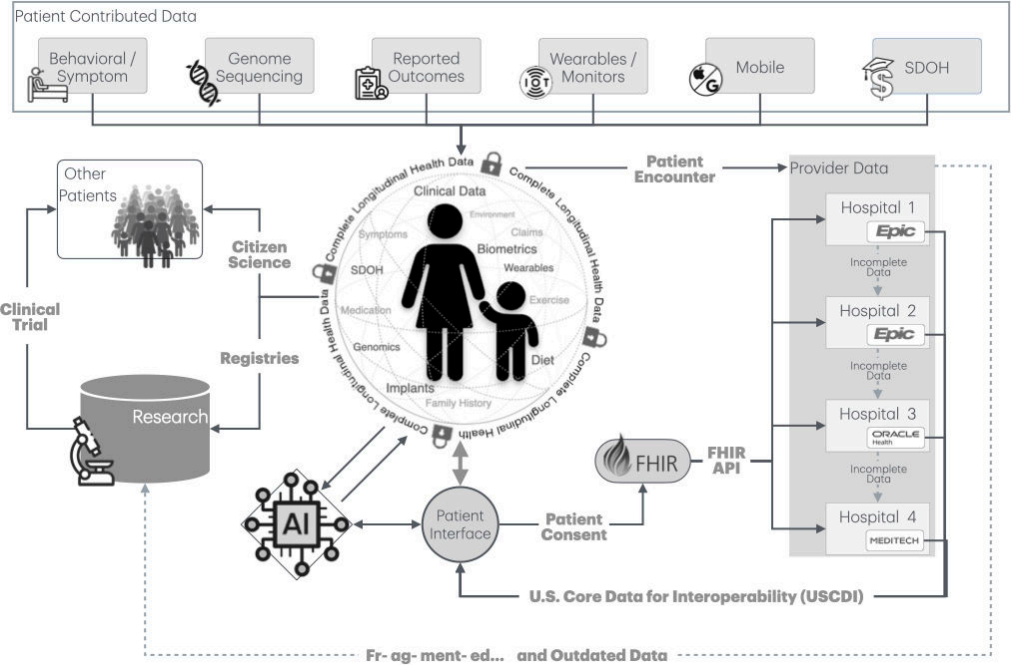
Sociotechnical framework of consumer-driven longitudinal health data aggregation for precision and translational medicine. AI: artificial intelligence; API: application programming interface; FHIR: Fast Healthcare Interoperability Resource; SDOH: social determinants of health.

The following highlight steps to pilot AI-enabled LHRs (Figure 5):

1. Demonstrate the future universal participatory behavior. Despite variation in cognitive ability, creativity, and ambition, all individuals share the common and basic responsibility of aggregating and granting access to their health data.
2. Attend to health IT standards and policies. It is critical to identify gaps in access, accuracy, completeness, and enforcement of longitudinal health data. Aggregating LHRs for every participant in a congenital genetic rare disease registry will reveal limitations and discrepancies of legacy “point-in-time” frameworks (eg, C-CDA for HIM requests) and the real-time FHIR-enabled interoperability standard, as promoted by the ASTP.
  - Leverage the 21st Century Cures Act Electronic Health Information (EHI) export rule. The EHI Final Rule, effective December 31, 2023, requires providers, health IT developers, and EMR vendors to ensure their systems can export complete EHI in a human- and machine-readable format.
  - EHI exports additional data beyond what is included in the USCDI, such as full protected narrative notes including progress notes, consultation notes, discharge summaries, and other unstructured documentation; billing and claims information; Digital Imaging and Communications in Medicine (DICOM); complete medical history, including past surgeries, inactive medications, discontinued allergies, and previous diagnostic results; device and implant data; and detailed provenance information, such as the data source and the time it was recorded.
3. Ensure data harmonization to leverage comprehensive LHRs. Rare disease registries are perfectly aligned to demonstrate harmonizing nonclinical and wearable data with multiple EMR data sources. For example, storing harmonized LHRs in a graph database using nodes and edges will allow for semantic queries of data for both humans and machine algorithms to provide better clinical care and research.
4. Optimize the value of using LLMs in health care. With comprehensive LHRs for an entire disease cohort, investigators can feed AI robust, real-world datasets to provide insights to complement their pretrained open data. This will help providers and researchers gain maximum insight and explore the upper limits of AI value. Knowing all available data are being considered frees providers to quickly iterate LLM prompt queries. Society can realize both limitations and power AI to augment clinical reasoning.
5. Establish a human custodian interface. Knowing consumers are at the center of translational science, the registry will provide a participant-focused (custodianship) interface. This can support data aggregation, a pipeline to experts, and research—enhanced by LLM analytics personalized for each user. A participant experience feedback loop will ensure optimal and ongoing participant engagement.
6. Formalize data ownership and consent frameworks. The participant interface will have a beneficial consent, access, and data ownership framework. This not only provides precision medicine capability for practitioners and research insights but also fuels access to data for unaffiliated clinical trials and population health studies. Ultimately, a data chain-of-ownership infrastructure will reshape the landscape and speed of research and drug discovery.
7. Rare disease registry proof of concept. Participants in rare disease registries serve as early adopters, using individual EHI exports for a comprehensive view of each patient’s medical history, going beyond minimum data required by the USCDI. This approach ensures that all relevant patient information is accessible. Applying this method within a rare genetic registry can help ASTP shape future versions of the USCDI and support enforcement of information blocking policies.

## Where to Start?

Of the approximately 7000 rare diseases recognized by the US Food and Drug Administration [38], less than 20% [39] have dedicated patient registries. Unlike the more common conditions, such as cystic fibrosis or muscular dystrophy, ultrarare communities tend to be tightly knit, with higher levels of intimate involvement and familiarity. Capitalizing on this advanced participatory behavior, a well-established, highly active registry for an existing ultrarare congenital genetic rare disease provides an ideal use case to demonstrate the power of LHR aggregation.

Diamond Blackfan Anemia (DBA) is a severe, chronic congenital blood disorder that affects fewer than 1 in 200,000 live births [40]. Caused by 28 known gene deletions that impair red blood cell production in the bone marrow, treatments include blood transfusions, stem cell transplantation, and corticosteroid therapies.

The Diamond Blackfan Anemia Registry (DBAR) [41], established in 1993 by Dr. Jeffrey Lipton [42] and Dr. Adrianna Vlachos [43] and operated at Northwell Health, is a premier example of a reputable rare disease infrastructure. Boston Children’s Hospital, St. Jude Children’s Hospital, Memorial Sloan Kettering Cancer Center, Children’s Hospital of Philadelphia, and the University of Chicago Medical actively contribute to DBA research and treatment. These prominent institutions (and collaboration with other major research centers) bring vital expertise in genetic mutation, bone marrow failure syndromes, hematologic malignancies, pediatric oncology, stem cell transplantation, and gene therapies, making them valuable partners in DBA treatment strategies and novel discoveries.

The DBAR is unique in the rare disease registry realm, with an exclusive focus on collecting disease-specific longitudinal and genetic data. Like CFFPR, DBAR’s narrow attention allows for a deep understanding of a single disorder; however, DBAR serves a smaller patient population, making participation in the community more personal and likely to occur. DBAR collaborates closely with the Diamond Blackfan Anemia Foundation (DBAF) [44], founded in 1994, to engage patients and families throughout the registry and research process.

The DBAF plays a critical role in raising awareness, connecting patients with expert providers, promoting initiatives to improve care and outcomes, and advancing research funding. They are a pioneering example of patient advocacy for rare disease, encouraging family participation in research and sharing real-world lived experiences. DBAR and DBAF’s strong reputation in patient engagement fosters widespread participation and ensures that all efforts fully align with their community’s needs and priorities.

DBA’s severe and ultrarare environment offers a perfect opportunity to demonstrate emerging consumer-driven, participatory behaviors and prototype the aggregation of comprehensive LHRs. With a long history of trust and success, the DBAR and DBAF registries and networks provide a manageable sized cohort and setting to pilot data aggregation strategies, identify technology and policy needs, and demonstrate how LLMs can advance DBA knowledge and shorten the diagnostic odyssey for undiagnosed patients.

## Significant Impact

By perfecting an LHR data aggregation framework—including nonclinical data, wearable inputs, and high-fidelity genomic tools, such as nanopore sequencing [45], and equipping DBAR with foundational and disease-specific LLMs, we can expedite insights into congenital hypoplastic anemias. More importantly, this refined DBAR-LHR model offers a scalable blueprint for all rare diseases, enabling dramatic improvements in outcomes, accelerating research, and potentially reducing the $1 trillion annual health care burden associated with rare conditions.

## Major Shift

If we accept the principle that comprehensive data improves outcomes [46], we must also recognize that patients and caregivers are the only consistent provenance (source) and legal owners of Longitudinal Health Records. No health care system, business interest, or government entity will generate LHRs comprehensively. Thus, the pathway to leverage 60 Zettabytes of health data already collected for greater diagnostic accuracy and appropriate treatment depends upon consumer agency and AI collaboration.

## Immediate Focus

We have the technology and the tools. We have the policy. Now we must use participatory behavior. No one cares as much as patients! Proving and scaling this participatory model is the most urgent priority.

The adoption of consumer-led LHR aggregation for rare disease will spearhead the inevitable paradigm shift. The administrative, participatory behavior, and technical foundations pioneered here will form the sociotechnical infrastructure needed for general health management and chronic and rare disease care to follow—transforming a US $13.1 trillion [47] health care industry and the health of 8 billion people on the planet.

## Abbreviations

AI: artificial intelligence
ASTP: Assistant Secretary for Technology Policy
C-CDA: Consolidated Clinical Document Architecture
CFFPR: Cystic Fibrosis Foundation Patient Registry
DBA: Diamond Blackfan Anemia
DBAF: Diamond Blackfan Anemia Foundation
DBAR: Diamond Blackfan Anemia Registry
EMR: electronic medical record
FHIR: Fast Healthcare Interoperability Resource
HIPAA: Health Insurance Portability and Accountability Act
HL7: Health Level 7 International
LHR: longitudinal health record
LLM: large language model
ONC: Office of the National Coordinator for Health Information Technology
PCD: patient-contributed data
USCDI: United States Core Data for Interoperability
USMLE: United States Medical Licensing Examination
