# Correction: Consumer Data Is Key to Artificial Intelligence Value: Welcome to the Health Care Future

**DOI:** 10.2196/82984

**Published:** 2025-09-10

**Authors:** James P Cummings

**Affiliations:** 1 Participatory Health, 20 Grasmere Ave, Fairfield, CT, 06824, United States, 1 (212) 280-1600

In “Consumer Data is Key to Artificial Intelligence Value: Welcome to the Health Care Future” (J Particip Med 2025;17:e68261), five corrections were noted.

First, the author’s name was corrected from “James C” to read as “James P Cummings.”

Second, the author’s affiliation was changed to “Participatory Health, 20 Grasmere Ave, Fairfield, CT, 06824, United States, 1 (212) 280-1600”.

Third, in the “Forever on Call” section, a repetition of “comprehensive” was removed.

Fourth, in the “Where to Start?” section, this sentence:

The Diamond Blackfan Anemia Registry (DBAR) [41, established in 1993 by Dr. Jeffery Lipton...

Was changed to read as follows:

The Diamond Blackfan Anemia Registry (DBAR) [41], established in 1993 by Dr. Jeffrey Lipton...

Finally, under the second paragraph of the Lone Custodian section, a repetition of the following was removed: “different providers over their lifetime.”

The correction will appear in the online version of the paper on the JMIR Publications website, together with the publication of this correction notice. Because this was made after submission to PubMed, PubMed Central, and other full-text repositories, the corrected article has also been resubmitted to those repositories.

